# Supplementary material for: Characteristics predicting reduced penetrance variants in the high-risk cancer predisposition gene TP53
Source: HGG Adv. 2025 Jul 21;6(4):100484. doi: 10.1016/j.xhgg.2025.100484 (PMC12337872; doi:10.1016/j.xhgg.2025.100484)
Supplement: Document S1. Figures S1–S8 [file mmc1.pdf]

HGGA, Volume *6*

Supplemental information

Characteristics predicting reduced penetrance variants in the high-risk cancer predisposition gene *TP53*

Cristina Fortuno, Marcy E. Richardson, Tina Pesaran, Kelly McGoldrick, Paul A. James, and Amanda B. Spurdle

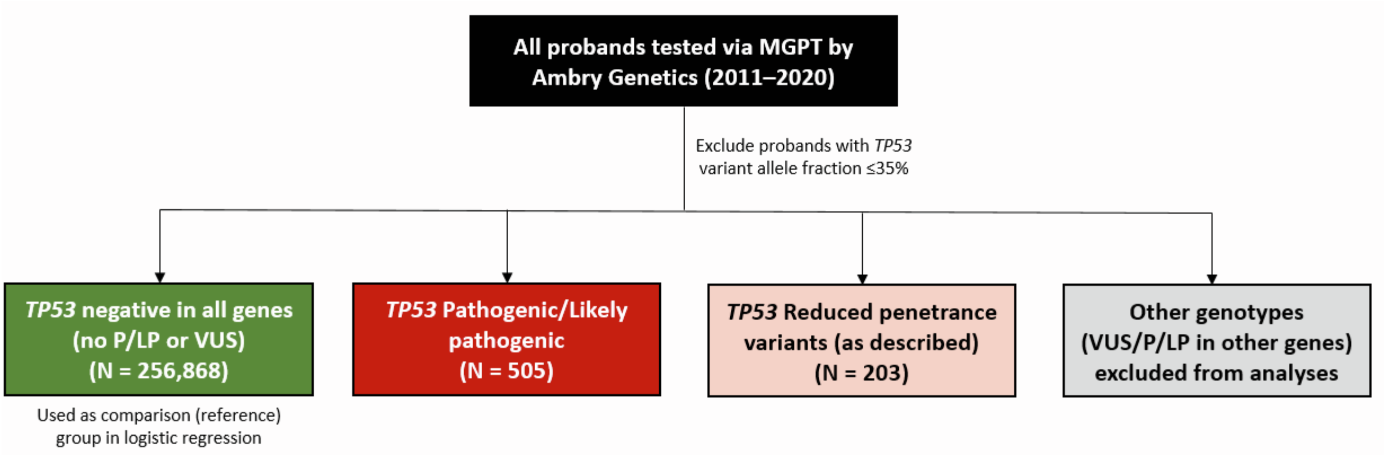

Supplemental Figure 1. Derivation of analytic cohorts from the Ambry Genetics proband dataset

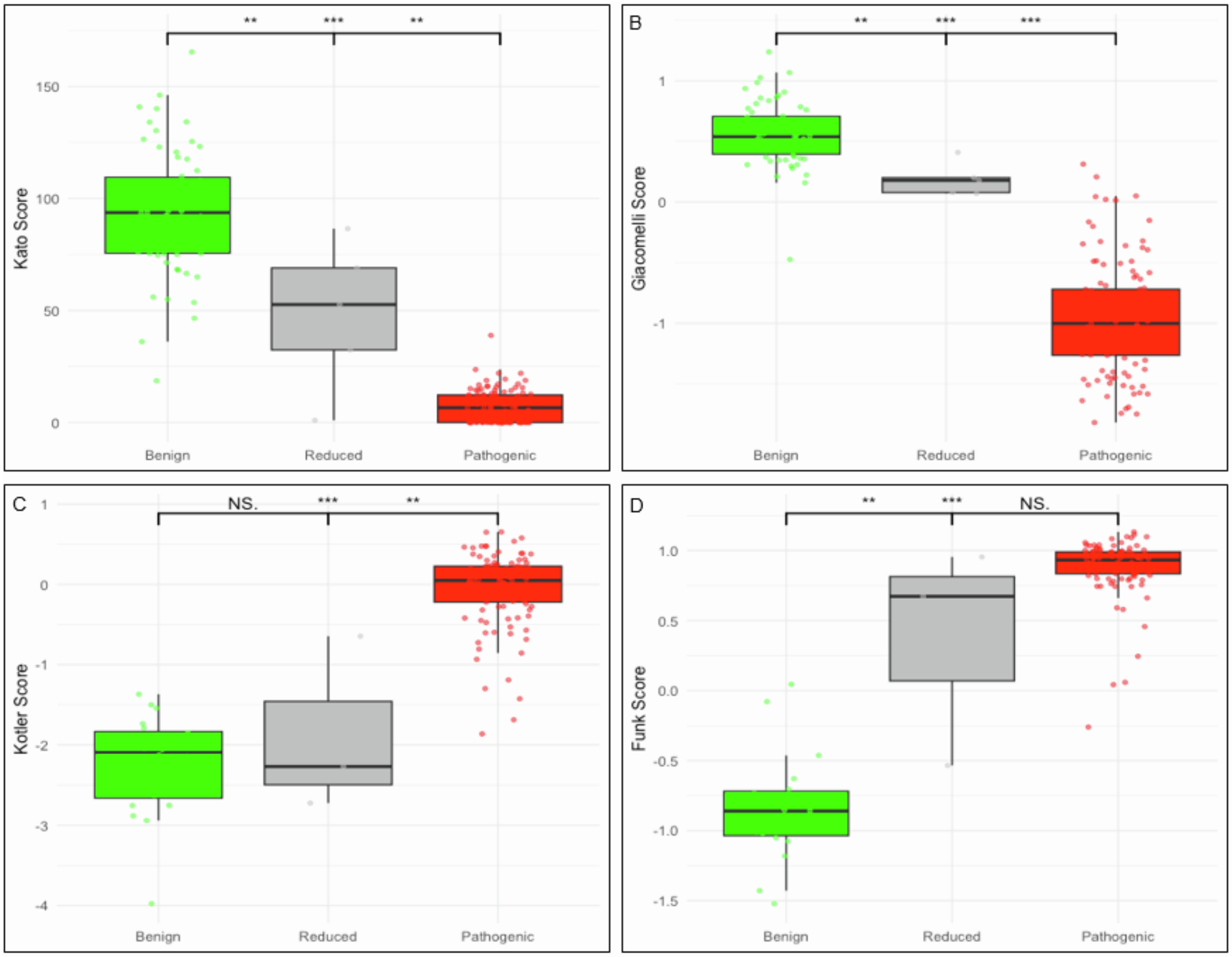

Supplemental Figure 2. Sensitivity analysis for the distribution of functional assay scores across *TP53* benign (green), reduced penetrance (grey), and pathogenic (red) variants. A: Kato et al., 2003 ($p = 1.21 \times 10^{-27}$), B: Giacomelli et al., 2018 ($p = 7.30 \times 10^{-28}$), C: Kotler et al., 2018 ($p = 1.27 \times 10^{-12}$), D: Funk et al., 2025 ($p = 1.52 \times 10^{-11}$). P-values refer to pairwise comparisons with Wilcoxon tests, where * = $p < 0.05$, ** = $p < 0.01$, and *** = $p < 0.001$. Statistical comparisons noted in the center are for Benign versus Pathogenic variants

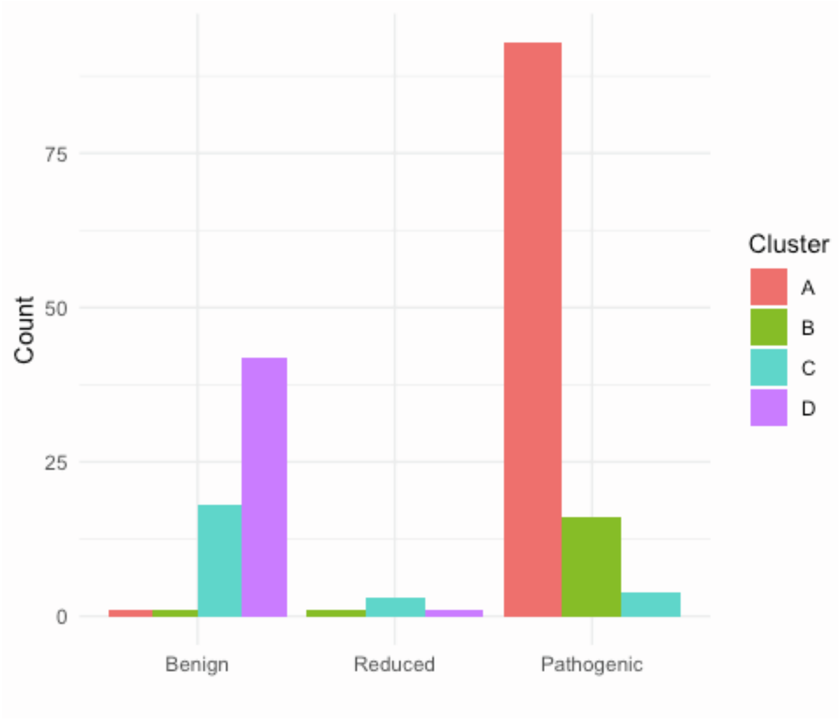

Supplemental Figure 3. Sensitivity analysis for the distribution of Kato clustering classes across *TP53* benign, reduced penetrance, and pathogenic variants (p = 1.40x10^{-32}). Classes represent a gradient of yeast-based transcriptional activity, from lowest (A) to highest (D) as per Montellier et al., 2024

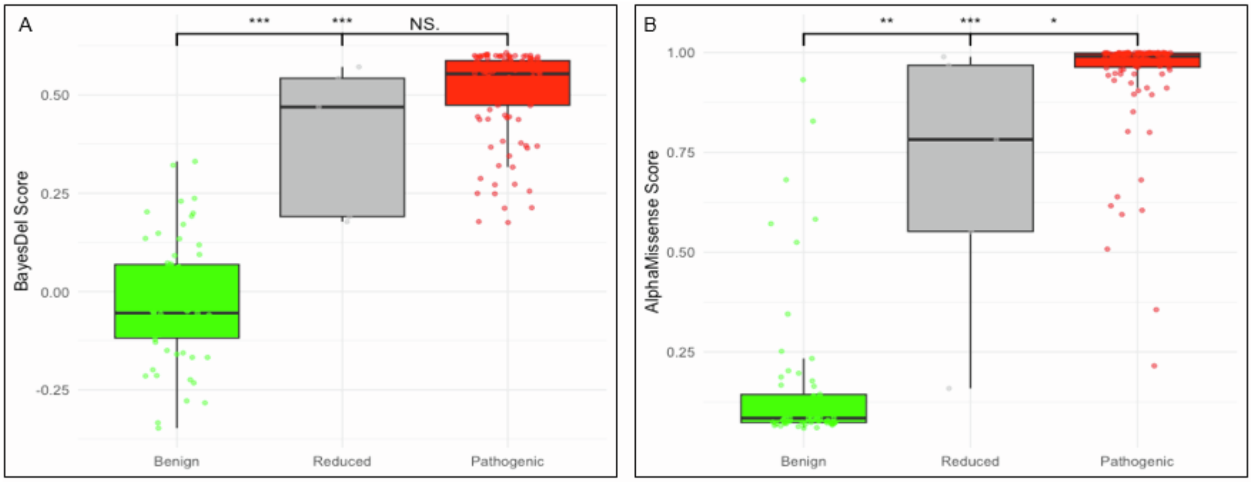

Supplemental Figure 4. Sensitivity analysis for the distribution of bioinformatic scores across *TP53* benign (green), reduced penetrance (grey), and pathogenic (red) variants. A: BayesDel (Feng et al., 2017) ($p = 1.22x10^{-26}$, B: AlphaMissense (Cheng et al., 2023) ($p = 8.98x10^{-27}$). P-values refer to pairwise comparisons with Wilcoxon tests, where * = $p < 0.05$, ** = $p < 0.01$, and *** = $p < 0.001$. Statistical comparisons noted in the center are for Benign versus Pathogenic variants

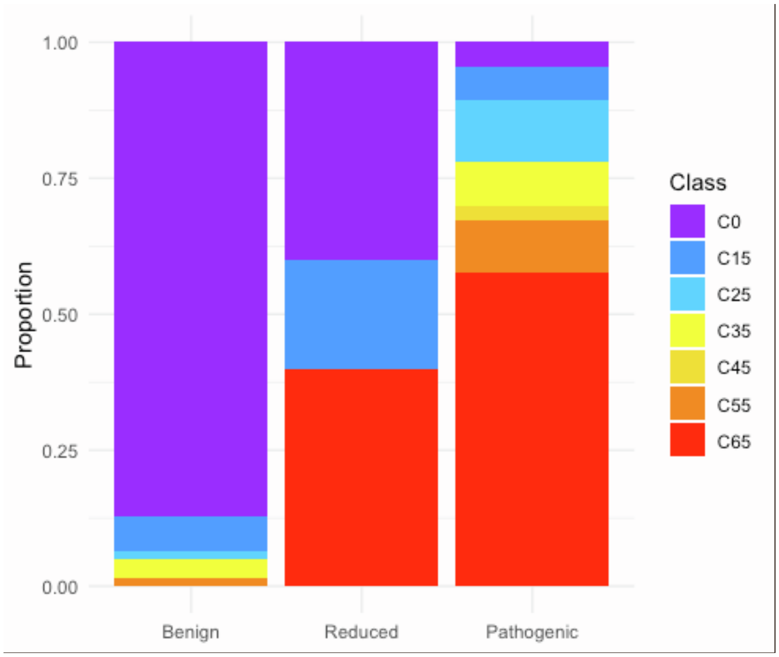

Supplemental Figure 5. Sensitivity analysis for the distribution of aGVGD categories across *TP53* benign, reduced penetrance, and pathogenic variants ($p = 3.75x10^{-22}$). C65 is predicted most likely to interfere with function, and C0 least likely, based on the biochemical variation at each position in a multiple sequence alignment of orthologous sequences as per Tavtigian et al., 2008

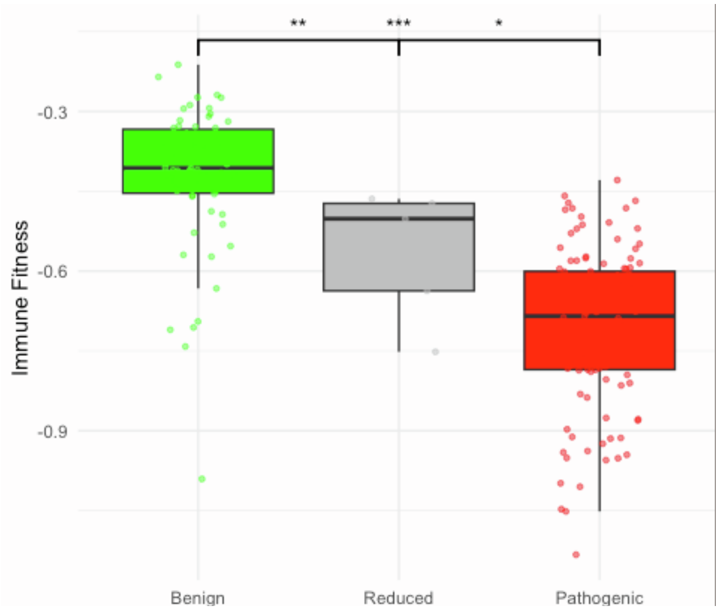

Supplemental Figure 6. Sensitivity analysis for the distribution of immune fitness across *TP53* benign (green), reduced penetrance (grey), and pathogenic (red) variant groups (Hoyos et al., 2022) ($p = 2.46x10^{-20}$). P-values refer to pairwise comparisons with Wilcoxon tests, where * = $p < 0.05$, ** = $p < 0.01$, and *** = $p < 0.001$. Statistical comparisons noted in the center are for Benign versus Pathogenic variants

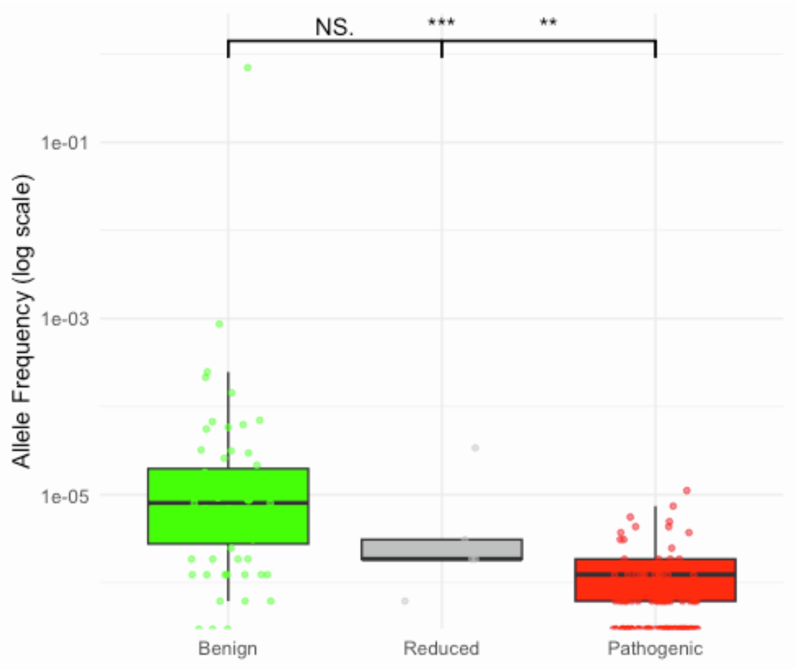

Supplemental Figure 7. Sensitivity analysis for the distribution of total allele frequency in gnomAD v4.1 across *TP53* benign (green), reduced penetrance (grey), and pathogenic (red) variant groups ($p = 2.89 \times 10^{-20}$). P-values refer to pairwise comparisons with Wilcoxon tests, where * = p < 0.05, ** = p < 0.01, and *** = p < 0.001. Statistical comparisons noted in the center are for Benign versus Pathogenic variants

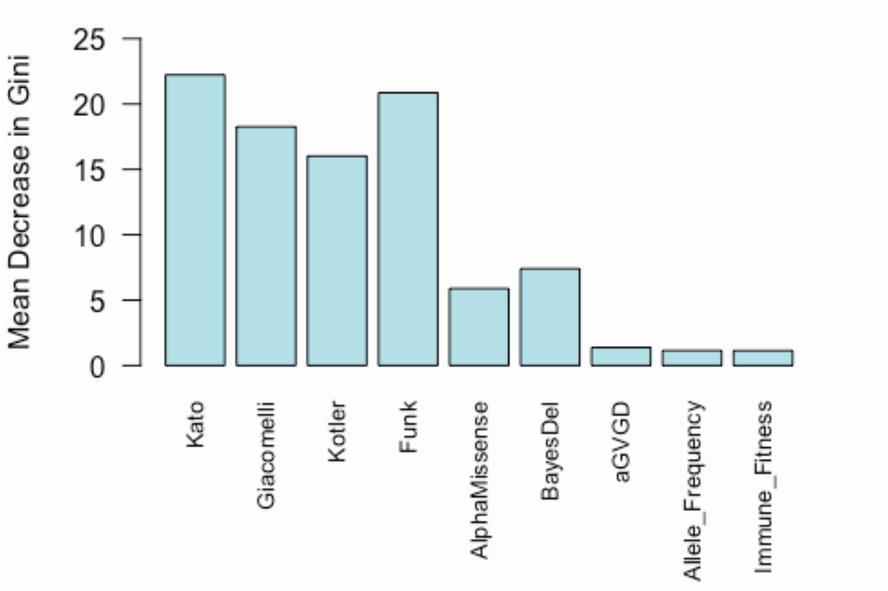

Supplemental Figure 8. Mean Decrease in Gini values representing the importance for the different components included in the random forest model to predict pathogenic, benign, and reduced penetrance variant status
